# Tracing the Origin of Genotype II African Swine Fever Virus in China by Genomic Epidemiology Analysis

**DOI:** 10.1155/2023/4820809

**Published:** 2023-03-31

**Authors:** Yong Zhang, Qinghua Wang, Zhongyi Zhu, Shujuan Wang, Shuyang Tu, Yongqiang Zhang, Yanli Zou, Yutian Liu, Chunju Liu, Weijie Ren, Dongxia Zheng, Yunling Zhao, Yongxin Hu, Lin Li, Chuan Shi, Shengqiang Ge, Peng Lin, Fengping Xu, Jinmin Ma, Xiaodong Wu, Hongchao Ma, Zhiliang Wang, Jingyue Bao

**Affiliations:** ^1^China Animal Health and Epidemiology Center, Qingdao 266032, China; ^2^Lars Bolund Institute of Regenerative Medicine, Qingdao-Europe Advanced Institute for Life Sciences, BGI-Qingdao, BGI-Shenzhen, Qingdao 266555, China; ^3^College of Life Sciences, University of Chinese Academy of Sciences, Beijing 518083, China; ^4^Pathogenesis Pharmaceutical Technology, BGI-Shenzhen, Shenzhen 518083, China; ^5^National Facility for Protein Science in Shanghai, Zhangjiang Laboratory, Shanghai Advanced Research Institute, Chinese Academy of Sciences, Shanghai 201210, China; ^6^BGI-Qingdao, BGI-Shenzhen, Qingdao 266555, China

## Abstract

The pandemic spread of African swine fever (ASF) has caused serious effects on the global pig industry. Virus genome sequencing and genomic epidemiology analysis play an important role in tracking the outbreaks of the disease and tracing the transmission of the virus. Here we obtained the full-length genome sequence of African swine fever virus (ASFV) in the first outbreak of ASF in China on August 3^rd^, 2018 and compared it with other published genotype II ASFV genomes including 9 genomes collected in China from September 2018 to October 2020. Phylogenetic analysis on genomic sequences revealed that genotype II ASFV has evolved into different genetic clusters with temporal and spatial correlation since being introduced into Europe and then Asia. There was a strong support for the monophyletic grouping of all the ASFV genome sequences from China and other Asian countries, which shared a common ancestor with those from the Central or Eastern Europe. An evolutionary rate of 1.312 × 10^−5^ nucleotide substitutions per site per year was estimated for genotype II ASFV genomes. Eight single nucleotide variations which located in MGF110-1L, MGF110-7L, MGF360-10L, MGF505-5R, MGF505-9R, K145R, NP419L, and I267L were identified as anchor mutations that defined genetic clusters of genotype II ASFV in Europe and Asia. This study expanded our knowledge of the molecular epidemiology of ASFV and provided valuable information for effective control of the disease.

## 1. Introduction

African swine fever (ASF) is a lethal contagious viral disease of domestic pigs and wild boars, which also affects African wild suits (warthogs and bushpigs) in an asymptomatic carrier state. Soft ticks serve as a natural reservoir and transmit the disease to the vertebrate host. African swine fever virus (ASFV) was first identified in Kenya in 1921. Since then, it has been reported in Africa, Europe, and South America [1]. In 2007, ASFV was introduced into the Georgian Republic and then swept across Europe and Asia [2–6]. In 2021, ASF was reported in Dominica and Haiti [7]. With no vaccine or any treatment available, affected pigs have been culled to try to contain the outbreaks. More than 50 countries are now affected by ASF, causing the death or culling of more than 9 million pigs. The pandemic spread of ASF has caused serious effects on the global pig industry, even leading to a critical global heparin shortage [8, 9].

ASFV is a large, encapsulated double-stranded DNA virus belonging to the *Asfivirus* genus of the *Asfarviridae* family. *Asfarviridae* family, together with the families *Mimiviridae, Pithoviridae, Marseilleviridae, Pandoraviruses, Phycodnaviridae, Iridoviridae*, and *Poxviridae*, composing the group of nucleocytoplasmic large DNA viruses (NCLDV), are also known as giant viruses [10]. The main primary target cells of ASFV belong to the monocyte/macrophage lineage. Upon ASFV infection, the highest titres are observed in tissues containing a large component of the mononuclear phagocyte system (reticuloendothelial cellular elements) like the spleen and lymph nodes [11]. Therefore, it is recommended in *African swine fever detection and diagnosis, a manual for veterinarians published by* the *FAO* that spleen and lymph nodes are the most important target organs being used to check for the presence of ASFV [12].

The genome of ASFV is a linear double-stranded DNA (dsDNA) molecule in the length of 171 kB to 193 kB with terminal inverted repeats and hairpin loops. It has a conserved central region (CCR) of about 125 kB, while the ends are variable in size. The left variable region and the right variable region contain different number of five multigene family (MGF) genes: MGF 100, 110, 300, 360, and 505/530, named after the average number of encoded amino acids. The CCR contains genes involving virus replication, assembly, and host cell function modulation. Phylogenetic analysis based on the partial sequence of the B646L gene (440 bp, nucleotide positions 103,630-104,069 of the ASFV genome) has revealed 24 genotypes of ASFV in Africa, from genotype I to genotype XXIIII. Genotype I is also endemic in Sardinia, Italy. ASFV strains recently circulating in Europe and Asia are classified as genotype II [5]. Several molecular markers have been used to typing genotype II ASFV in Europe and Asia [13–15]. However, the limited information provided by a partial genomic sequence was not sufficient for tracing the transmission of ASFV.

Genomic epidemiology analysis has been widely used to trace the transmission of RNA viruses such as SARS-CoV-2, Ebola, and Zika virus, which provided valuable information for the interpretation of field epidemiology data and the implementation of efficient control measures [16–19]. The development of next-generation sequencing (NGS) and third-generation sequencing techniques and hence the reduction of genome sequencing cost has made feasible large-scale sequencing for large dsDNA virus genomes [20, 21]. Genome sequence diversity analysis of 45 epidemiologically varied virus isolates has been conducted to reveal clues to the evolution of the variola (smallpox) virus [22]. Dynamic genome evolution and complex virocell metabolism of globally-distributed giant viruses was investigated by generating and analyzing 501 metagenome-assembled genomes of NCLDV from environments around the globe [10]. A curated dataset has recently been constructed, containing a total of 123 ASFV genome sequences including 64 genotype I genomes, 42 genotype II genomes, and 17 genomes belonging to other genotypes [23]. The genome-wide genome similarity between ASFV genotype I and genotype II ranges between 82.4–94.7%. A few molecular epidemiology studies based on genome sequences have been conducted to reconstruct the evolution of ASFV globally or to trace the origin and evolution of genotype I ASFV in Sardinia [24–26]. Up to date, the evolutionary dynamics of genotype II ASFV, currently widespread in Eurasia remain unclear.

In China, the first outbreak of ASF was reported on August 3^rd^, 2018 in Liaoning province. The causative strain of this outbreak was identified as ASFV genotype II by molecular characterization based on the partial sequence of the B646L gene [2]. However, the full-length genome sequence of the ASFV that caused the first outbreak of ASF in China was not available. The first published genome sequence of ASFV in China was generated from field samples collected in an ASF outbreak on September 2^nd^, 2018 in Anhui province [27]. From then on, genome characterization of ASFV strains circulating in China has been described. To trace the origin of ASFV in China, it is essential to determine the complete genome sequence of ASFV in the first outbreak.

In this study, we generate a novel full-length genome sequence of ASFV from clinical samples collected in the first outbreak of ASF in China and assess the genome sequence diversity of genotype II ASFV in China. In addition, we present phylogenetic analysis to demonstrate that genotype II ASFV has evolved into different genetic clusters since being introduced into Europe and then Asia. Moreover, we identify anchor mutations that define the genetic clusters of genotype II ASFV. We verify a strong monophyletic relationship among all the ASFV genome sequences from China and other Asian countries, which share a common ancestor with those from Central or Eastern Europe. Our study serves as an example of how genomic epidemiology can be used to trace the origin of ASF and track the transmission of the virus. Our findings also provide insight into the genomic evolution of genotype II ASFV during the ongoing pandemic in Europe and Asia, highlighting its important role in controlling this lethal swine disease.

## 2. Materials and Methods

### 2.1. Genome Sequencing and Genome Sequence Comparison

On August 3^rd^, 2018, an outbreak of ASF was reported in a swine farm in Shenbei district, Shenyang city in Liaoning province. Eight infected pigs were sampled for diagnosis at the infected farm. Spleen, lymph node, and kidney samples were collected from each pig and sent to our laboratory for confirmation. Clinical samples were screened with real-time PCR with amplification targeting the B646L gene. The ASFV China/LN/2018/1 strain was grouped into genotype II by partial sequencing of the B646L gene. For each pig, the sample with the lowest Ct value in the ASFV real-time PCR assay was further used for genome sequencing. Genome sequences were determined directly from the clinical samples to avoid cell-culture-driven sequence variation, as previously reported [27]. An identical ASFV genome sequence was found in samples from 8 pigs. Therefore, the ASFV genome from the spleen of a 6-month-old infected pig was used in this study to represent the first ASF outbreak in China. Nine genotype II ASFV genomes described in a recently published curated dataset were used for genome wide diversity analysis on genotype II ASFV in China. These genomes represented genotype II ASFV collected in China from September 2^nd^, 2018 to October 1^st^, 2020 (Table 1).

### 2.2. Genome Sequences Alignment and Recombination Analysis

A total of forty-two genotype II ASFV genomes described in a curated dataset were used for phylogenetic analysis [23]. The genome of LR812933.1/Arm/07/CBM/c2 was not included in this study for fear of possible contamination. The final dataset included a total of 41 genotype II ASFV genomes collected from 15 countries from 2007 to 2020. The GenBank accession number, the year, and the country of isolation were listed in Table 1. Multiple nucleotide alignments of genome sequences was conducted using the MAFFT software (version 7.475) [45]. The alignment of ASFV genomes was analyzed for recombination using seven different recombination detection methods (RDP, GENECONV, BootScan, MaxChi, Chimaera, SiScan, and 3Seq).

### 2.3. Phylogenetic Analysis

Maximum likelihood (ML) phylogenetic trees were estimated by RAxML v8.2.12 [46] using GTR + G nucleotide substitution model. ML bootstrapping was performed with 1000 resamples to assess the robustness of tree topologies. The final tree was midpoint rooted by FigTree v1.4.2. (https://tree.bio.ed.ac.uk/software/figtree/). Bayesian inference of phylogeny was performed using the Bayesian Markov Chain Monte Carlo (MCMC) method in the BEAST package 1.8.2 [47]. The most suitable substitution models were selected in JModelTestv2.1.10 [48]. The dataset was analyzed using the GTR + G substitution model. The Markov Chain Monte Carlo chains were run for 2 × 10^8^ generations and sampled every 2000 generations. TreeAnnotator v1.10.4 was used to generate maximum-clade credibility (MCC) trees with a burn-in rate of 10%. Summary phylogenies were visualized in FigTreev1.4.2. Phylogenetic network analysis was performed with the Network software (version 10.2), which is available as shareware on the Fluxus Technology website (https://www.fluxus-engineering.com/) [49]. Mutations of single nucleotide variations (SNVs) in the coding region sequences of ASFV were used. The data was run by median joining network algorithm and Steiner tree algorithm, respectively, with the epsilon parameter set to zero. We also performed an exploratory run by setting the epsilon parameter to 10. It is revealed that both algorithms yielded an identical structure of network.

### 2.4. The Analysis of Evolutionary Rates and Estimation of Time to the Most Recent Common Ancestry

Temporal dynamics of ASFV were analyzed with time-resolved phylogenies using the Bayesian MCMC method in the BEAST package 1.8.2. The datasets were analyzed using the GTR + G substitution model under a lognormal uncorrelated relaxed clock model with a coalescent constant size model. The MCMC analyses were run in duplicate for 2 × 10^8^ generations, sampling every 2000 generations. Convergence was assessed from the effective sample size (ESS) with a 10% burn-in using Tracer v1.6. ESS values above 200 were accepted.

### 2.5. Phylogeographic Analysis

The spatial transmission patterns of ASFV were reconstructed using a phylogeographic analysis in BEAST 1.8.2. The geographic location and sampling time of all ASFV genome sequences were obtained as described previously. The best-supported pairwise diffusions were identified using SPREAD3 v0.9.6 [50]. Bayesian Stochastic Search Variable Selection (BSSVS) was used to estimate the significance of pairwise switches between trait states. Migration pathways with a Bayes factor greater than 5 and a mean posterior value greater than 0.5 were considered to be important.

### 2.6. Detection of Selection Pressures

Positive selection was detected using Datamonkey (https://www.datamonkey.org/). Single Likelihood Ancestor Counting (SLAC), Fixed Effects Likelihood (FEL), Mixed Effects Model of Evolution (MEME), and Fast Unconstrained Bayesian AppRoximation (FUBAR) were used to infer selection [51]. Sites identified by at least two algorithms were considered to be undergoing conservative positive selection.

### 2.7. Protein Structure Prediction

The three-dimensional structure of protein I267L was modelled using the Alphafold2 program [52] and PyMOL 1.4.1 [53].

## 3. Results

### 3.1. Genome Sequences Diversity of Genotype II ASFV in China during 2018 to 2020

We obtained the full-length genome sequence of the ASFV strain for the first outbreak of ASF in China in Shenyang, Liaoning province on August 3^rd^, 2018. The spleen sample collected from an ASF-confirmed pig on the infected farm was used for next-generation sequencing. The final assemble of the ASFV China/LN/2018/1 genome was achieved from a reference-based alignment consisting of 149,069 mapped reads (0.27% of 54,956,232 total reads) with an average depth of 85 reads per nt. The ASFV China/LN/2018/1 whole-genome sequence has a length of 189,397 bp, not including terminal inverted repeats and cross links. The sequence data has been submitted to the GenBank database under accession number OP856591.

**Figure 1:**
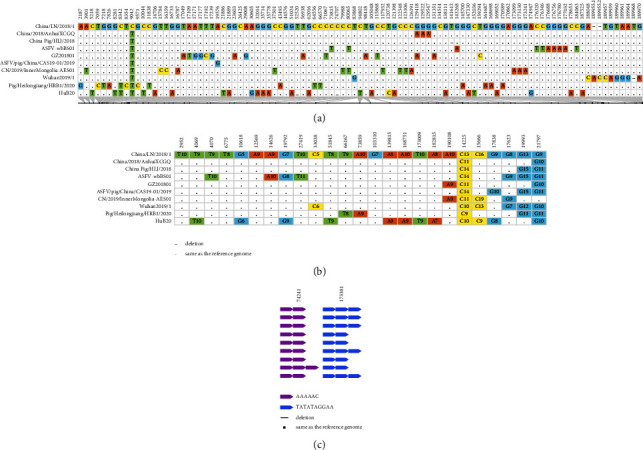
Genome sequence diversity of genotype II ASFV in China from August 3^rd^ to October 1^st^, 2020. Nucleotide position in the reference genome Georgia 2007/1 was shown. (a) The number and detail of mutations at SNPs and indel. (b) The number and detail of mutations of homopolymer and variable polyG/C sequence. (c) Sequence variations of the tandem repeat sequences (TRS). Each arrow with different color represents a type of TRS. The sequence of the repeat unit in each TRS was also shown.

The ASFV China/LN/2018/1 genome was added to the other 9 genotype II ASFV genomes in China in the curated dataset described previously, making a total of 10 ASFV genome sequences collected in China from August 3^rd^, 2018 to October 1^st^, 2020. The genomic diversity of the genotype II ASFV genomes in China was further investigated. Comparing to the ASFV China/LN/2018/1 genome, mutations in each genome sequence were checked manually. A total of 128 variable sites were found (Figure 1). Further investigation of these sites revealed 2 structural variations (in MW656282.1/Pig/Heilongjiang/HRB1/2020), 2 Tandem repeat sequences variations, 99 single nucleotide variations (SNVs), 19 homopolymer variations (1-2 bases of insertion and deletion in the homopolymeric tracts), and 6 highly variable poly G or poly C tract variations.

### 3.2. Phylogenetic Analysis of Genotype II ASFV and Estimation of Time to Most Recent Common Ancestry

The ASFV China/LN/2018/1 genome was added to other genotype II ASFV genomes in the curated dataset, making a total of 42 ASFV genome sequences for further analysis. Signatures of recombination were detected in 3 sequences (LS478113.1/Estonia 2014, MG939584.1/Pol16_20538_o9, MW656282.1/Pig/Heilongjiang/HRB1/2020) by at least five of the seven different detection algorithms (*P* < 0.01). These 3 genome sequences were not included in the further phylogenetic analysis. Considering the uncertainty of the terminal inverted repeat sequence on both genomic ends, the coding region sequence corresponding to the nucleotide site 2113-190,166 of the reference genome Georgia 2007/1 was used for phylogenetic analysis of the final dataset of 39 genotype II ASFV strains. These genomes were collected from domestic pigs (23 genomes) and wild boars (16) from 15 countries including Belgium (2), China (9), the Czech (1), Estonia (1), Georgia (1), Germany (1), Hungary (1), Lithuania (1), Malawi (1), Moldova (1), Poland (10), Russia (6), South Korea (1), Tanzania (1), Timor-Leste (1), and Ukraine (1) during 2007 to 2020 (Table 1).

**Figure 2:**
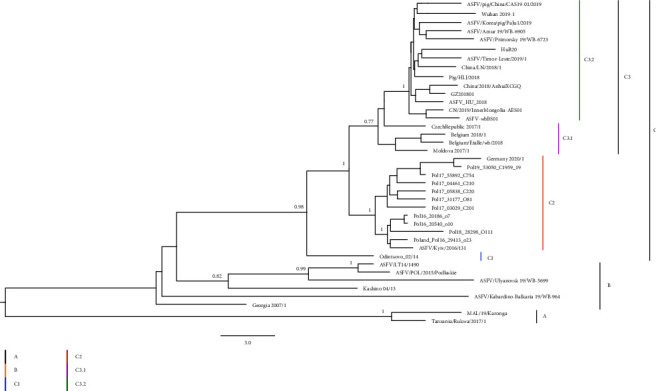
Maximum-clade credibility phylogenetic tree of genotype II ASFV genome sequences. The tree is midpoint-rooted. The scale bar is given in numbers of substitutions per site. The posterior probabilities higher than 0.7 are shown at the corresponding nodes. Cluster assignment is shown by color: black for clade A yellow for clade B blue for clade C1, orange for clade C2, purple for clade C3.1, and green for clade C3.2.

The maximum clade credibility (MCC) phylogenetic dendrogram of ASFV was constructed by the Bayesian-based coalescent approach. The MCC tree showed 3 clades with temporal and spatial correlation (Figure 2). Clade A included two strains collected from Africa. Clade B included 7 strains which were collected from Georgia, Russia (3 strains), Lithuania, and Poland from 2007 to 2019. Clade C included strains collected from Europe and Aisa from 2014 to 2020, grouping into one singleton (subcalde C1) and two subclades (C2 and C3). Subcalde C1 was a singleton of Odintsovo_02/14, a strain collected from Russia in 2014. Subcalde C2 included 13 strains: 11 strains collected from Poland during 2016 to 2019, one strain from Germany in 2020, and one strain from Ukraine in 2016. Subclade C3 was composed of 2 clusters, cluster C3.1 and Cluster C3.2. Cluster C3.1 included 4 strains from the Czech Republic, Moldova, and Belgium from 2017 to 2018. Cluster C3.2 was composed of one strain from Hungary in 2018, 9 strains collected from China from 2018 to 2020, one strain from South Korea in 2019, one strain from Timor-Leste in 2019 and two strains from eastern Russia in 2019. The tree topology indicated that genotype II ASFV has evolved into different genetic groups since being introduced into Europe and then Asia.

**Figure 3:**
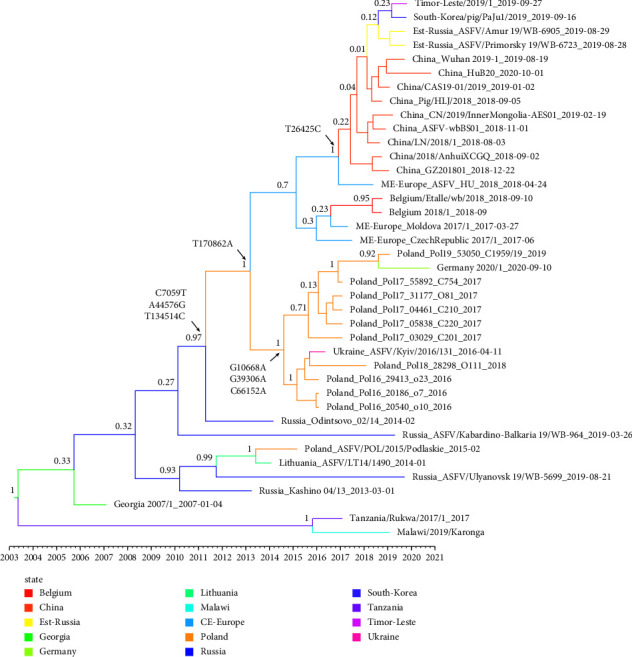
Time-revolved phylogenetic tree based on genotype II ASFV genome sequences using Bayesian MCMC analysis. The tree was estimated using the GTR + G substitution model under a lognormal uncorrelated relaxed clock model with a coalescent constant size model. Branches are color-coded by country or region. The scale bar indicates the time in year. The posterior probability is shown at each node.

The estimates of evolutionary rates and time to most recent common ancestry (TMRCA) of genotype II ASFV were calculated by the Bayesian-based coalescent approach. The mean evolutionary rate of the ASFV GII genome was estimated at 1.312 × 10^−5^ nucleotide substitutions per site per year (95% HPD, 7.7432 × 10^−6^–1.8733 × 10^−5^). The divergence times and the countries of origins of genotype II ASFV were estimated by using the Bayesian phylogenetic analysis. For clade B, which is expanding in Europe and Asia, the median TMRCA was estimated to be August 2005 (95% HPD: April 2000–January 2007), and its country of origin was inferred as Georgia, with strong support (100% posterior probability) (Figure 3).

### 3.3. Subgroup Divergence of Genotype II ASFV in China and Estimation of Time to Most Recent Common Ancestry

Sequences collected from different provinces in China during 2018 to 2020 were widely interspersed, with no particular grouping according to distinct temporal and spatial correlation. Sequences from other neighboring countries, including South Korea, Timor-Leste, and Eastern Russia, were embedded within the cluster of 9 genomes obtained from China. There was strong support for monophyletic grouping of all the sequences from Asia and a strain from Hungary which was sampled on April 24^th^, 2018 (100% posterior probability). Similar phylogenetic relationships for these genomes were also seen using maximum likelihood (ML) phylogenetic reconstruction (Figure S1). There was also strong support for grouping sequences from Asia, Hungary, Belgium, Czech, Moldova, Germany, Poland, and Ukraine (100% posterior probability). It is suggested that all the ASFV genotype II genomes in China share a single origin and have a common ancestor with those from Central or Eastern Europe.

TMRCA estimation showed that the emergence of the ancestor strain of ASFV in Asia dated back to April 2017 (95% HPD, February 2016; March 2018) (Figure 3). The ancestor at the node linking all the Asian ASFV sequences with the most closely related sequence in Hungary was dated to October 2016. It is consistent with the 5-month time difference between the first outbreak in Hungary in April 2018 and the first outbreak in China in August 2018. The median TMRCA of the genomes in the other countries in Asia was July 2018 (95% HPD, June 2017; April 2019). It is consistent with the 13-month time difference between the first outbreak in China in August 2018 and the first outbreaks in South Korea and Timer-Leste in September 2019.

### 3.4. Phylogeographic Analysis of Genotype II ASFV

**Figure 4:**
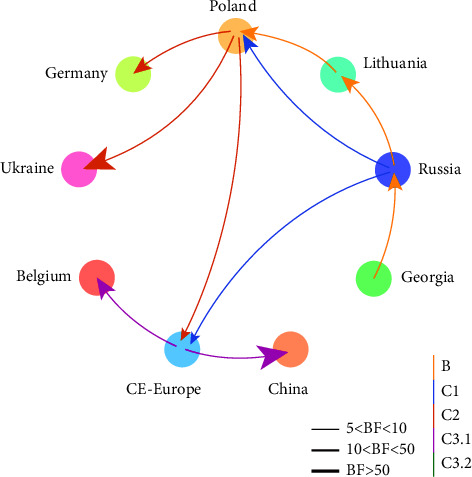
Spatial diffusion pathway of genotype II ASFV in Europe and Asia. Arrows indicate the direction of the switch; only switches supported by BF values >5 are shown. Line thickness is proportional to the dispersal route significance level. The line is colored according to the cluster assignment.

To further investigate the origin of genotype II ASFV in China, a discrete phylogeographic analysis was conducted. The Bayesian stochastic search variable selection (BSSVS) method was used to infer the geographic spread (Figure 4). It was revealed that ASFV spread from Central or Eastern Europe into China was strongly supported with a Bayes factor (BF) of 64.86 and an associated posterior probability (PP) of 0.82. Well-supported rates were also identified for ASFV spread from Poland to Ukraine (BF = 81.39; PP = 0.85), from Poland to Germany (BF = 38.44; PP = 0.73), and from Central or Eastern Europe to Belgium (BF = 40.14; PP = 0.74). It is suggested that phylogeographic analysis based on high-quality genome sequences could be used for tracing the transmission of ASFV during an epidemic.

### 3.5. Identification of Anchor Mutation in Genotype II ASFV Genomes

**Figure 5:**
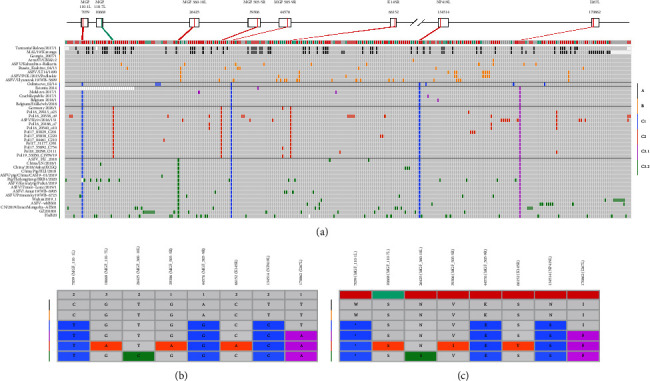
Pattern of genotype II ASFV single nucleotide polymorphisms (SNPs) distribution. (a) Distribution of SNPs in genotype II ASFV genomes. A total of 277 SNPs show a pattern of genotype II ASFV mutation accumulation, showing one mutation site per column. The strain name is shown at the left, indicating one strain per row. The grey blocks indicate sequence identity with the reference genome Georgia 2007/1 sequence. The top row shows the type of mutation (dark grey, intergenic; green, synonymous; red, nonsynonymous), with the gene location indicated above. Cluster assignment is shown at the left by color. (b) Distribution of 8 anchor SNPs in different cluster of genotype II ASFV genomes. The top row shows the codon position of SNPs with the gene location indicated above. (c) Distribution of amino acid changes of anchor SNPs in different cluster of genotype II ASFV genomes. The top row shows the type of mutation (green, synonymous; red, nonsynonymous), with the gene location indicated above.

To trace the occurrence and possible fixation of mutations during evolution of genotype II ASFV, we investigated the SNVs in ASFV genotype II genomes. All the SNVs were extracted and listed in a matrix (Figure 5(a)). Eight SNVs were identified as anchor mutations, which were fixed in all the sequences in corresponding clusters. The gene location and codon position of each anchor mutation was also displayed (Figure 5(b)). The distribution of amino acid changes of each anchor mutation was also investigated (Figure 5(c)). Sequences in clade C were distinguished from those in clade B by three nonsynonymous mutations: C7059T (G590A/Trp197*∗*in MGF110-1L), A44576G (A967G/Lys323Gluin MGF505-9R) and T134514C (A1241G/Asn414Ser in NP419L). Sequences in subclade C2 and C3 shared one nonsynonymous mutation, T170862A (A583T/Ile195Phe in I267L). Subclade C2, including genomes from Poland, Ukraine, and Germany, was defined by one synonymous and two nonsynonymous mutations: G10668A (C60T/Ser20 in MGF110-7L), G39306A (G988A/Val330Ile in MGF505-5R), and C66152A (C434A/Ser145Tyr in K145R). A nonsynonymous mutation in MGF360-10L (A986G/Asn329Ser), T26425C, characterized cluster C3.2 containing genomes from Hungary, eastern Russia, China, and other Asian countries, as shown in Figure 3.

**Figure 6:**
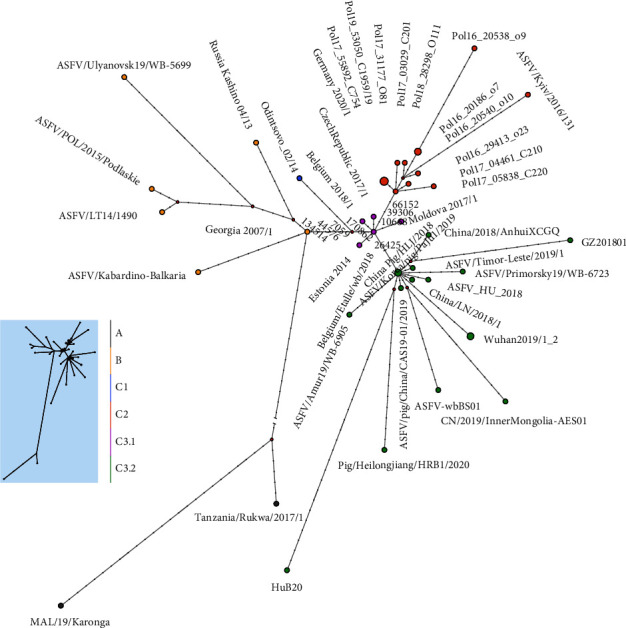
Phylogenetic network of genotype II ASFV genomes. The network was constructed from the SNPs in the coding region of the genome sequences. The circle nodes represent ASFV strains, which are proportional to the number of taxa. Each notch on the links represents a mutation event. Red dots indicate putative ancestor nodes. Cluster assignment is shown by color. The median-joining network algorithm and the Steiner algorithm were used.

A median joining phylogenetic network was constructed based on the SNVs for exploring evolutionary relationships among ASFV genotype II genomes. Three clades with temporal and spatial correlation were identified (Figure 6). The putative ancestor of clade C was derived from that of clade B by 4 anchor mutations. The putative ancestor of subclade C2 was derived from the common node by 3 anchor mutations, and cluster C3.2 one anchor mutation. Mutational branches in cluster C3.2 formed a star-like radiation topology, indicating a single origin and then expansion of strains in this cluster. Two sequences were identified as the ancestral node in cluster C3.2, one from Heilongjiang province in China on September 5^th^, 2018 and the other from South Korea on September 16, 2019. A total of 10 mutational branches radiated from the ancestral node of cluster C3.2, representing 9 genomes collected from China during 2018 to 2020, one genome from Hungary, one genome from Timor-Leste, and two genomes from eastern Russia.

### 3.6. Measure of Selection Pressures in Genotype II ASFV Genomes

Site-specific selection pressures were measured as the ratio of nonsynonymous (dN) to synonymous (dS) nucleotide substitutions per site to infer sites subjected to positive selection in the coding sequences of MGF110-1L, MGF110-7L, MGF360-10L, MGF505-5R, MGF505-9R, K145R, NP419L, and I267L. Using a posterior probability of 0.88 for FUBAR, 12 sites were identified as under positive selection. Using the default threshold of significance of *P* < 0.1, two sites were found by SLAC method. The threshold was reevaluated manually into *P* < 0.05 to check for the accuracy of the default parameters. The same result was achieved by the SLAC method. Amino acid 414 in NP419L and amino acid 195 in I267L were identified as under positive selection by both the FUBAR and SLAC methods (Table 2).

**Figure 7:**
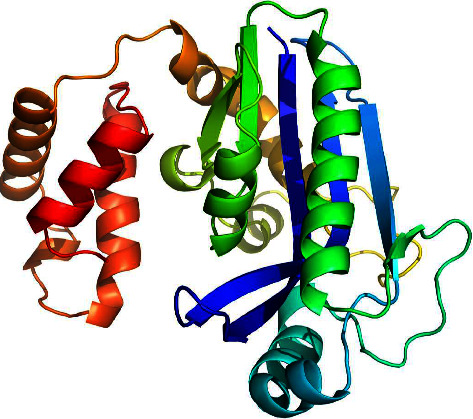
The three-dimensional structure modelling of I267L by the Alphafold2 program.

The three-dimensional structure of I267L was modelled using the Alphafold2 program (Figure 7). The function of I267L was predicted to be binding with nucleic acids.

## 4. Discussion

To our knowledge, this is the first in-depth analysis on genotype II ASFV genomic evolution. Our estimation of the evolutionary rate of 39 ASFV genotype II genomes collected in Eurasia and Africa between 2007 and 2020 is 1.312 × 10^−5^ nucleotide substitutions per site per year (95% HPD, 7.7432 × 10^−6^; 1.8733 × 10^−5^), which is around 3.9 times faster than the value calculated for ASFV genotype I genomes collected in Sardinia [24, 26]. The widespread presence of ASFV in Eurasia, the large population of infected domestic pigs and wild boars, and the inadequate biosecurity of the pig industry in Asia may have sped the expansion and evolution of the virus.

The presence of a single putative common ancestor for all ASFV genome sequences in China provides clear evidence for a single introduction of the virus. According to the analysis of currently available genome sequences, ASFV in China shares the same origin with those in Hungary, Belgium, the Czech, Moldova, Germany, Poland, and Ukraine. However, it is not possible for us to ascribe the countries of origin for genotype II ASFV in China to a country or an area in a country. There is a data gap for those countries which were not represented by ASFV genome sequences or had a limited number of ASFV genome sequences published. Between 2007 and August 2018, a total of 11 European countries reported ASFV outbreaks. Among these countries, genome sequences from Romania and Belarus are not publicly available. Genome sequences of ASFV in Russia, especially the Far East, Ukraine, Lithuania, Latvia, and Estonia between 2014 and 2018 are limited. Previous studies demonstrated that the sequencing of more ASFV samples dramatically improved the resolution of ASFV phylogenetic structure and permitted finer molecular dating [24]. More genome sequences of ASFV strains circulating in European countries will likely help improve our knowledge on the molecular epidemiology of genotype II ASFV.

In this study, we construct the transmission pathway of the spread of ASFV genotype II basing on the genome-wide SNVs. The putative-ancestor-virus-centered radial shape of the Asian cluster suggests that samples collected from different provinces in China and other Asian countries may come from the same virus origin. According to the field epidemiology investigation, long-distance transmission of animal or animal products played an important role in the spread of ASFV in Asia [54, 55]. Not only it is true that more than half of the world's domestic pigs are raised in Asia, but also it has the highest population density of farmed pigs in the world. The pig production in Asia, in general, was not well industrialized had a inadequate biosecurity [9]. It explains how the ASF virus rapidly swept Asia in a short time after the introduction and produced a variety of mutational variants.

The anchor SNVs which defined clusters of ASFV GII in Europe and Asia were located in MGF110-1L, MGF110-7L, MGF360-10L, MGF505-5R, MGF505-9R, K145R, NP419L, and I267L. K145R and MGF505-5R have been used as molecular markers to differentiate ASF viruses circulating in Poland [14]. MGF-360-10L, MGF-505-9R, and I267L have also been used to differentiate ASF viruses collected in Russia [41]. However, this is the first time to identify 8 anchor mutations for differentiating genotype II ASFV. These anchor mutations can serve as candidate molecular markers for genetic characterization of genotype II ASF viruses circulating in Europe and Asia and help trace the origin and transmission of this virus.

The function of some of the genes harboring these anchor mutations has been determined. As shown in Table 3, 3 out of the 8 genes have been previously identified as involving immune modulation of host responses. MGF 110-7L induces host cell translation suppression and stress granule formation by activating the PERK/PKR-eIF2 alpha pathway [57]. MGF360-10L has been implicated in the modulation of the type I interferon (IFN) response and associated with the virulence of the virus [58]. I267L acts as an important virulence factor by inhibiting RNA polymerase III-RIG-I-mediated innate immunity [61]. Although the function of MGF505-5R and MGF505-9R remains unknown, it is well known that MGF530/505 genes have roles in suppressing the induction of type I IFN [58]. Previous studies revealed that pK145R is a highly abundant viral protein that accumulates diffusely in the cytoplasm of infected cells but is not essential for the in vitro propagation of ASFV [59]. MGF110-1L is also nonessential for in vitro propagation [56]. The mutations in these genes might be necessary for the adaptation of ASFV to the large population of domestic pigs in Europe and Asia.

ASFV is replicated and assembled in swine macrophage cells, which are rich in free oxygen radicals and cause constant damage to the virus genome. To efficiently overcome these DNA damages, ASFV virus has evolved its own repair system, including repair DNA polymerase (AsfvPolX) and ligase (NP419L, also known as AsfvLIG). AsfvLIG is one of the most error-prone ligases identified to date, which plays important roles in the strategic mutagenesis and genotype formation of ASFV. AsfvLIG contains one N-terminal region, one adenylation domain in the center, and one OB-fold domain (OB) at the C-terminus [60]. The site residue subjected to positive selection (Asn414Ser) locates in the OB-fold domain, in close proximity to two active site residues (Leu402 and Gln403 of the OB domain) which are crucial for the catalytic efficiency of AsfvLIG. Therefore, it is most likely that the positive selection of DNA ligase may facilitate the mutagenesis of the ASFV genome and further adaption of the virus to pandemic spread in Eurasia.

Our study suggests that the structure of the evolution topology for genotype II ASFV strains is determined by SNVs. The role of other mutation events in ASFV evolution needs to be further investigated. ASFV strains with structural variations including recombination, fragment deletion, or insertion are of specific interest to researchers because of the potential for virulence attenuation. Previous research on genome sequence comparisons of different ASFV genotypes suggested that recombination might play an important role in ASFV evolution [62–64]. According to our analysis of genotype II ASFV strains, two structural variations are identified as clade A specific. The role of structural variations in the evolution of genotype II ASFV needs to be further investigated.

## 5. Conclusion

ASFV is presently causing a global animal health emergency because of its devastating effects on pig populations. The lack of vaccines or effective therapies against ASFV urges the need to address the existing relevant gaps in knowledge of the virus genome evolution. Genomic epidemiology has played an irreplaceable role in investigating the origin, tracing the spread, and monitoring the evolution of ASFV. It will play a key role in helping contain the ASFV pandemic's spread.

## Figures and Tables

**Table 1:** List of genotype II ASFV genome sequences used in this study.

NCBI Acc nos.	Strains	Hosts	Lengths	Collection dates	Countries	Genotypes	Ref
LR536725.1	Belgium 2018/1	Wild boar	190,599	2018/9	Belgium	II	[28]
MK543947.1	Belgium/Etalle/wb/2018	Wild boar	190,202	2018/9/10	Belgium	II	[29]
	China/LN/2018/1	Pig	189,397	2018/8/3	China	II	This study
MK128995.1	China/2018/AnhuiXCGQ	Pig	189,393	2018/9/2	China	II	[27]
MK333180.1	Pig/HLJ/2018	Pig	189,404	2018/9/5	China	II	[30]
MK645909.1	ASFV-wbBS01	Wild boar	189,394	2018/11/1	China	II	NA
MK940252.1	CN/2019/InnerMongolia-AES01	Pig	189,403	2019/2/19	China	II	NA
MN172368.1	ASFV/pig/China/CAS19-01/2019	Pig	189,405	2019/1/2	China	II	[31]
MN393476.1	Wuhan 2019-1	Pig	190,576	2019/8/19	China	II	NA
MT496893.1	GZ201801	Pig	189,393	2018/12/22	China	II	NA
MW521382.1	HuB20	Pig	188,643	2020/10/1	China	II	NA
MW656282.1	Pig/Heilongjiang/HRB1/2020	Pig	189,355	2020/9/12	China	II	[32]
LR722600.1	CzechRepublic 2017/1	Wild boar	190,594	2017/6	Czech	II	[33]
LS478113.1	Estonia 2014	Wild boar	182,446	2014/9	Estonia	II	[34]
FR682468.2	Georgia 2007/1	Pig	190,584	2007/1/4	Georgia	II	[35]
LR899193.1	Germany 2020/1	Wild boar	190,592	2020/9/10	Germany	II	NA
MN715134.1	ASFV_HU_2018	Wild boar	190,601	2018/4/24	Hungary	II	[36]
MK628478.1	ASFV/LT14/1490	Wild boar	189,399	2014/1	Lithuania	II	[37]
MW856068.1	MAL/19/Karonga	Pig	183,325	2019	Malawi	II	[38]
LR722599.1	Moldova 2017/1	Pig	190,598	2017	Moldova	II	[20]
MG939583.1	Pol16_20186_o7	Pig	189,401	2016	Poland	II	[39]
MG939584.1	Pol16_20538_o9	Pig	189,399	2016	Poland	II	[39]
MG939585.1	Pol16_20540_o10	Pig	189,405	2016	Poland	II	[39]
MG939586.1	Pol16_29413_o23	Pig	189,393	2016	Poland	II	[39]
MG939587.1	Pol17_03029_C201	Wild boar	189,405	2017	Poland	II	[39]
MG939588.1	Pol17_04461_C210	Wild boar	189,401	2017	Poland	II	[39]
MG939589.1	Pol17_05838_C220	Wild boar	189,393	2017	Poland	II	[39]
MH681419.1	ASFV/POL/2015/Podlaskie	Wild boar	189,394	2015/2	Poland	II	[40]
MT847620.1	Pol17_55892_C754	Pig	189,414	2017	Poland	II	[14]
MT847621.1	Pol18_28298_O111	Pig	189,409	2018	Poland	II	[14]
MT847622.1	Pol17_31177_O81	Pig	189,422	2017	Poland	II	[14]
MT847623.2	Pol19_53050_C1959/19	Pig	189,413	2019	Poland	II	[14]
KJ747406.1	Kashino 04/13	Wild boar	189,387	2013/3/1	Russia	II	NA
KP843857.1	Odintsovo_02/14	Wild boar	189,333	2014/2	Russia	II	NA
MT459800.1	ASFV/Kabardino-Balkaria 19/WB-964	Wild boar	189,252	2019/3/26	Russia	II	NA
MW306190.1	ASFV/Amur 19/WB-6905	Wild boar	189,248	2019/8/29	Russia	II	[41]
MW306191.1	ASFV/Primorsky 19/WB-6723	Wild boar	189,256	2019/8/28	Russia	II	[41]
MW306192.1	ASFV/Ulyanovsk 19/WB-5699	Wild boar	189,263	2019/8/21	Russia	II	[41]
MT748042.1	ASFV/Korea/pig/PaJu1/2019	Pig	190,597	2019/9/16	South Korea	II	NA
LR813622.1	Tanzania/Rukwa/2017/1	Pig	183,186	2017	Tanzania	II	[42]
MW396979.1	ASFV/Timor-leste/2019/1	Pig	192,237	2019/9/27	Timor-Leste	II	[43]
MN194591.1	ASFV/Kyiv/2016/131	Pig	191,911	2016/4/11	Ukraine	II	[44]

**Table 2:** List of amino acid positions identified as evolving under positive selection.

Protein	MEME	FUBAR	FEL	SLAC
I267L	—	195 (0.84)	—	195
		129 (0.83)		
		229 (0.81)		
K145R	—	145 (0.87)	—	—
MGF110_1L	—	—	—	—
MGF110_7L	—	39 (0.88)	—	—
MGF360_10L	—	329 (0.87)	—	—
MGF505_5R	—	330 (0.86)	—	—
		338 (0.86)		
		462 (0.87)		
MGF505_9R	—	323 (0.87)	—	—
		465 (0.87)		
NP419L	—	414 (0.87)	—	414

The default threshold of significance (*P* < 0.1) was used for MEME, SLAC, and FEL. Posterior probability of 0.81∼0.88 was used for FUBAR. Posterior probability was given in parentheses. “—” indicates no positively selected sites was identified.

**Table 3:** List of function of the proteins harboring these anchor mutations.

Genes	Functions	References
MGF 110-1L	Nonessential for in vitro propagation and nonaffect viral virulence	[56]
MGF 110-7L	Induces host cell translation suppression and stress granule formation by activating the PERK/PKR-eIF2 alpha pathway	[57]
MGF 360-10L	Implicated in the modulation of the type I interferon (IFN) response and associated to the virulence of the virus	[58]
MGF 505-5R	NA	NA
MGF 505-9R	NA	NA
K145R	A highly abundant viral protein accumulated diffusely in the cytoplasm of infected cells but nonessential for in vitro propagation of ASFV	[59]
NP419L	DNA ligase	[60]
I267L	An important virulence factor by inhibiting RNA polymerase III-RIG-I-mediated innate immunity	[61]

## Data Availability

The genome sequence data generated in this research has been deposited in the GenBank database under accession number OP856591.
