# Supplementary material for: Tracing the Origin of Genotype II African Swine Fever Virus in China by Genomic Epidemiology Analysis
Source: Transbound Emerg Dis. 2023 Mar 31;2023:4820809. doi: 10.1155/2023/4820809 (PMC12017148; doi:10.1155/2023/4820809)
Supplement: Supplementary Materials — Figure S1 to this article can be found as supplementary material. [file 4820809.f1.docx]

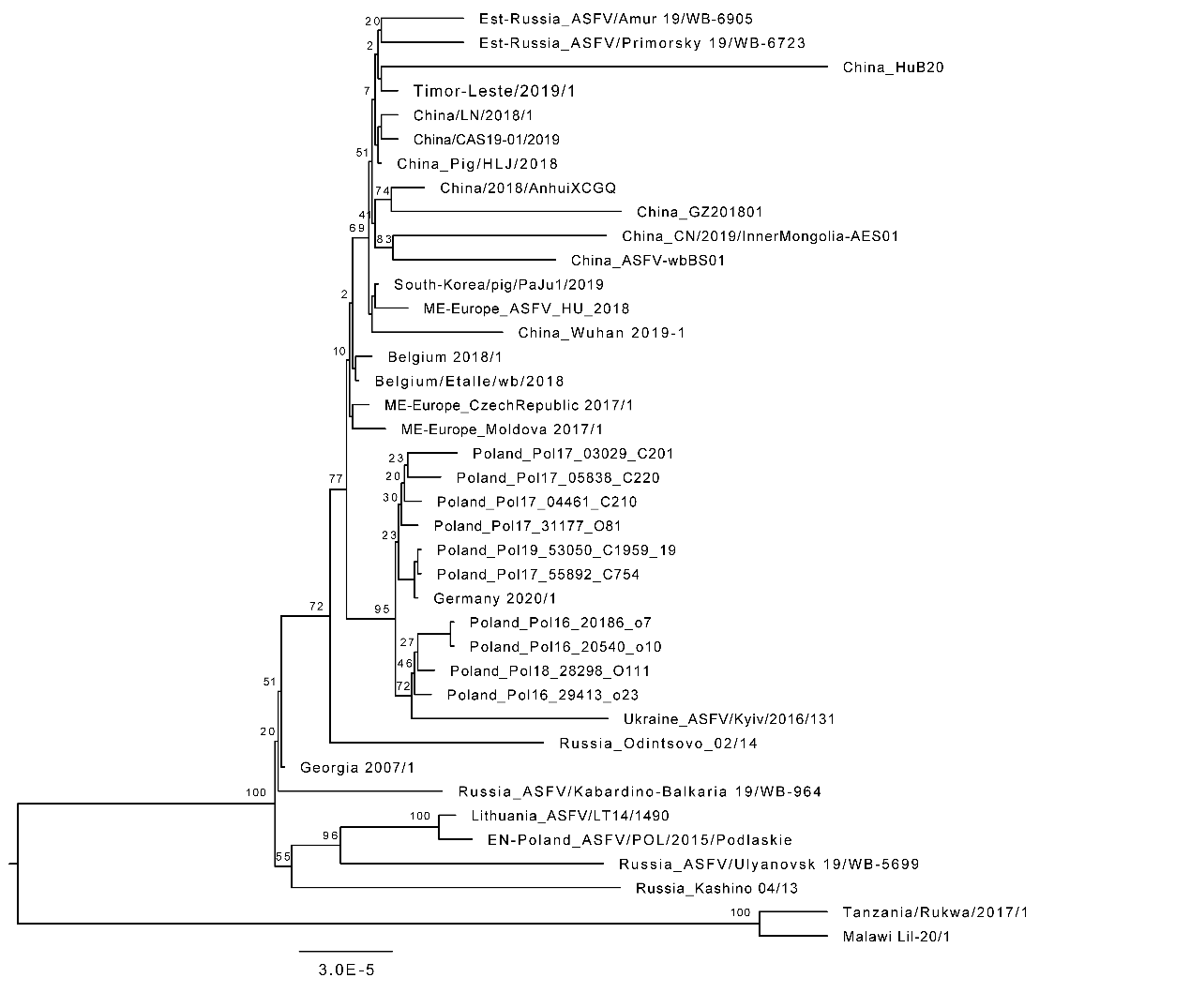


Figure S1. Maximum likelihood phylogenetic tree of ASFV genome sequences. The tree is midpoint unrooted. The scale bar is given in numbers of substitutions per site. Bootstrap resampling (1,000 replications) support values are shown at the nodes.
